# The Relationship Between Infertility Stress and Awareness/Utilization of Benefits Under the ‘Family and Youth Support Law’ Among Iranian Infertile Couples: A Cross‐Sectional Study

**DOI:** 10.1002/hsr2.72578

**Published:** 2026-05-27

**Authors:** Saber Azami‐Aghdash, Ramin Rezapour, Samin Banaei Rezaeiyeh, Zahra Vakilazad Sarabi, Leyla Najafi, Mohsen Nouri, Esmail Ezzati

**Affiliations:** ^1^ Medical Philosophy and History Research Center Tabriz University of Medical Sciences Tabriz Iran; ^2^ Tabriz Health Services Management Research Center Tabriz University of Medical Sciences Tabriz Iran; ^3^ Student Research Committee Tabriz University of Medical Sciences Tabriz Iran; ^4^ Faculty of Management and Medical Information Sciences Tabriz University of Medical Sciences Tabriz Iran; ^5^ Health Management and Economics Research Center, Health Management Research Institute Iran University of Medical Sciences Tehran Iran; ^6^ Naghadah School of Nursing Urmia University of Medical Sciences Urmia Iran

**Keywords:** infertility, stress, “Support for the Family and Youth of the Population” law

## Abstract

**Background and Aims:**

Infertility is recognized as one of the major stressors in life, with health, psychological, social, and cultural consequences. The aim of this study was to assess the relationship between infertility‐related stress and awareness and follow‐up of receiving benefits from the “Support for the Family and Youth of the Population “ law among infertile couples in East Azerbaijan province, Iran.

**Methods:**

This cross‐sectional correlational study was conducted in the city of Tabriz during the years 2023–2024. The participants were Iranian infertile couples of reproductive age (15–50 years) who had visited healthcare centers providing infertility services in East Azerbaijan Province. Data were collected using the Newton Infertility Stress/Problems Questionnaire and a researcher‐made questionnaire assessing awareness, perceived importance, and follow‐up of facilities related to the ‘Family Support and Youthful Population Law.’ SPSS version 16 and STATA version 17 software were used to analyze the data.

**Results:**

This study included 396 participants (response rate: 94.2%). The total infertility stress score averaged 169.78, indicating a relatively high level of stress among infertile couples. Correlation analysis revealed that infertility stress was significantly associated with all examined variables except age and duration of marriage. Spearman correlation analysis showed that the strongest correlations were observed with sexual concerns (*r* = 0.74, 95% CI: 0.69–0.78, *p* < 0.01) and relationship concerns (*r* = 0.73, 95% CI: 0.68–0.77, *p* < 0.01). Additionally, age showed a significant association with awareness and follow‐up (*r* = 0.14). Furthermore, multiple linear regression analysis identified the strongest predictors of infertility stress as, in descending order: the need for parenthood (B = 2.579), relational concerns (B = 1.464), and social concerns (B = 1.254).

**Conclusion:**

The findings suggest that while increased awareness, perceived importance, and pursuit of supportive facilities might ostensibly reduce stress, in practice, they are paradoxically associated with higher stress levels, which may be related to unrealistic expectations and bureaucratic barriers. This finding underscores the necessity for a fundamental revision of supportive policies and their implementation strategies.

## 1 Introduction

Infertility is currently a significant global public health and social issue [1]. It is defined as the inability of a couple to achieve pregnancy after 1 year of regular unprotected sexual intercourse [2, 3]. Worldwide, an estimated 10%–15% of couples are affected by infertility [4], with approximately 8%–12% of all couples globally experiencing infertility, indicating that one in every ten couples faces primary or secondary infertility [5, 6]. Beyond being a medical condition, infertility can profoundly impact well‐being and quality of life [7, 8]. It is ranked as one of the most significant life stressors, carrying psychological, social, and cultural consequences. For instance, infertile patients often experience higher levels of anxiety, depression, and stress [9, 10, 11].

Women's health throughout their lifespan, particularly during reproductive years, is a priority for healthcare systems worldwide. Indicators of reproductive and maternal health serve as critical markers of societal health and developmental progress [12]. Furthermore, population structure plays a pivotal role in a nation's socio‐political authority and advancement across multiple domains. Thus, formulating and implementing effective policies to foster economic, political, social, and cultural growth is not only vital but also an indispensable pillar for achieving any nation's overarching goals [13].

In Iran, declining population growth rates and aging demographics pose major social, political, healthcare, and economic challenges [14, 15]. Additionally, high infertility rates constitute a significant population and health concern [16]. A large‐scale 2005 study on infertility prevalence in Iran revealed that nearly a quarter (24.9%) of Iranian couples experienced primary infertility at some point in their marital life [17]. Given Iran's aging population and worsening demographic crisis, the “ Support for the Family and Youth of the Population” law was enacted in 2021. This policy aims to encourage marriage and childbearing by offering financial and logistical support. Given that one of the main target groups in this law is infertile couples, many facilities and programs have been prepared in this regard. Since one of the main and most key factors influencing couples to obtain information and receive these facilities is the level of perceived problems and stress due to infertility, it is necessary to measure the level of perceived problems and stress due to infertility and its relationship or impact on obtaining information and pursuing receipt of information and services through the “Support for the Family and Youth of the Population” law, and ultimately the impact of this group of people in society as a key part of this law, be examined. Because, based on the results of the literature search and the experiences of researchers, there is no evidence and documentation available that indicates the measurement of the relationship between stress and problems due to infertility and awareness and pursuit of receiving facilities from the “Support for the Family and Youth of the Population” law among infertile couples. Therefore, there is a need to scientifically and accurately examine the relationship between these two concepts and positions. For this purpose, the present study, which is a correlational study, was designed and implemented to measure the relationship between infertility‐related stress and awareness and follow‐up of receiving benefits from the “ Support for the Family and Youth of the Population “ law among infertile couples in East Azerbaijan province, Iran.

## 2 Methods

### 2.1 Study Design

**Figure 1:**
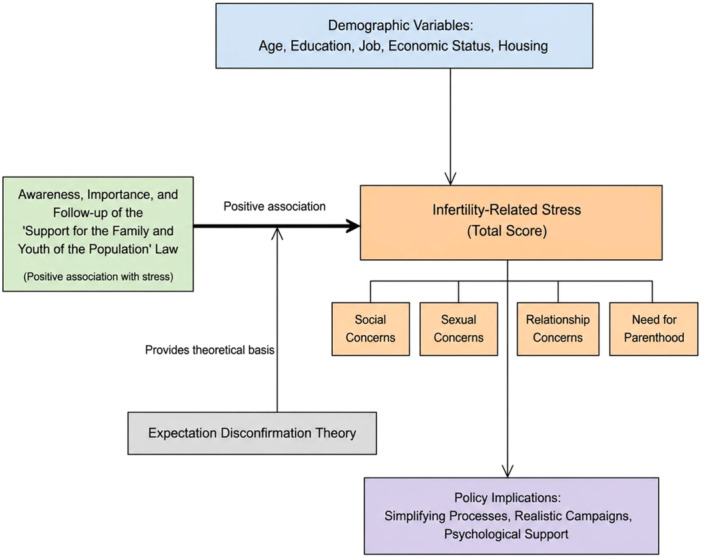
Conceptual framework of the study. Demographic variables (age, education, job, economic status, housing) were entered as candidate predictors. Awareness, perceived importance, and follow‐up of the “Support for the Family and Youth of the Population” law showed a positive correlation with infertility‐related stress (total score and subscales: social, sexual, relationship concerns, and need for parenthood). Expectation Disconfirmation Theory provides the theoretical basis. The framework also summarizes key policy implications derived from the findings: simplifying administrative processes, realistic awareness campaigns, and integrating psychological support alongside financial incentives.

The present study is a correlational study conducted in Tabriz City in 2023 and 2024. Tabriz is the capital of East Azerbaijan province in northwestern Iran. The city covers an area of 245 square kilometers and is the fourth largest city in Iran with a population of approximately 1.8 million [18]. The conceptual framework of the study is summarized in Figure 1.

### 2.2 “Support for the Family and Youth of the Population” Law

The Law on the Protection of the Family and Youthful Population was enacted in Iran with the objective of increasing fertility rates and counteracting demographic decline. The primary target groups of this legislation include young couples, particularly newlyweds, and families with at least one child. To mitigate socioeconomic barriers to childbearing, the law introduces a range of incentives, such as extended maternity leave (9 months for women and 2 weeks for men), low‐interest marriage and childbirth loans, tax exemptions for larger families, employment priority for mothers with children, and housing and healthcare subsidies. Additionally, the policy incorporates educational and cultural support measures, including media campaigns and family counseling programs, aimed at fostering attitudinal shifts to facilitate the realization of population rejuvenation policies.

### 2.3 Participants, Sample Size, and Sampling Method

The participants in this study were individuals from infertile couples (i.e., one or both partners) of reproductive age (15–50 years) who had visited health centers providing infertility services in East Azerbaijan Province. The inclusion criteria were: diagnosis of infertility (based on the scientific definition) for at least one partner; prior use of infertility services at East Azerbaijan health centers for ≥ 3 months; willingness to participate; marriage status; Iranian citizenship; and basic literacy. Exclusion criteria included: non‐Iranian residents in Iran; single individuals; and unwillingness to cooperate with the study.

The required sample size was estimated based on a power analysis for correlation coefficients. Assuming a two‐tailed test with *α* = 0.05, power = 0.80, and a conservative medium effect size (*r* = 0.3) for infertility‐related stress, the minimum required sample size was calculated as 84 participants. However, to account for the complex nature of the study (multiple subgroup analyses and regression modeling), we aimed for a larger sample. Using the approach suggested by Tabachnick and Fidell [19], for regression analyses (N > 50 + 8 m, where m is the number of predictors), with up to 15 candidate predictors, the required sample size would be at least 170 participants. Considering the infertility prevalence rate (~ 13%) and to account for potential attrition, we initially targeted 420 participants. The final sample of 396 participants far exceeds the minimum requirements for the planned statistical analyses.

### 2.4 Unit of Analysis

Individual participants were treated as the unit of analysis. Although data were collected from couples, the final sample included a larger proportion of women (77.6%) and did not allow for paired or clustered analyses at the couple level due to the absence of matched data for both partners in most cases. Therefore, intra‐couple dependency was not modeled, which represents a limitation of the study.

### 2.5 Rationale for Convenience Sampling and Potential Biases

Convenience sampling was employed due to practical constraints in accessing infertile couples across multiple healthcare centers with diverse ownership types (governmental, private, military) and geographic distribution throughout East Azerbaijan Province. Random sampling would have required a comprehensive sampling frame of all infertile couples in the province, which does not exist due to the absence of a centralized infertility registry. To minimize selection bias, we implemented several strategies. First, all centers providing infertility services in the province were invited to participate. Second, within each center, trained interviewers approached all eligible couples consecutively during the data collection period (rather than selecting specific individuals). Third, data collection spanned multiple months (12 months) to capture seasonal variations. Nevertheless, convenience sampling may introduce bias: our sample may overrepresent individuals who are more motivated, have greater access to healthcare, or have higher health literacy. Additionally, couples with very high stress levels may have declined participation. Therefore, generalizability to the entire population of Iranian infertile couples should be made with caution, and future studies with probability sampling designs (e.g., cluster‐randomized or stratified sampling) are warranted.

### 2.6 Data Collection Tools

The tool used in this study includes two standard questionnaires for measuring stress/infertility problems by Newton et al. 1999 and a researcher‐made questionnaire on awareness, importance, and follow‐up of receiving facilities regarding the “ Support for the Family and Youth of the Population “ law. The first questionnaire is the Infertility Stress Questionnaire by Newton et al. which was designed and developed to measure infertility stress [20]. This questionnaire has 46 questions and includes 5 components: social concerns, sexual concerns, relationship concerns, childless lifestyle, and the need to be a parent, and measures infertility stress based on a six‐point Likert scale. This questionnaire was examined in Iran in 2011 by Latifnejad Rudsari et al. [21], and its content, face, and criterion validity were assessed as appropriate, and the Cronbach's alpha coefficient calculated for it was also estimated to be above 0.7.

The second questionnaire was designed and developed by the researcher [22]. To identify and list the questions of the initial questionnaire, first, 10 people with expertise in the field of the subject under study and known in the field of research in health sciences were consulted. After the interviews were conducted, the research team members immediately transcribed and analyzed the texts and statements of the people. The environment for these interviews was Tabriz University of Medical Sciences, related research centers, and organizations and bodies interested in the “ Support for the Family and Youth of the Population “ law. Based on the opinions of these people and a review of the initial texts on similar websites and articles, the initial questions of the questionnaire were extracted. Then, with the participation of students (8 people), professors (4 people), and ordinary people (13 people), a qualitative assessment of the face validity of the instrument was carried out. In this stage, determining the duration of completing the questionnaire, difficulty in understanding phrases and words, appropriateness and desirable relevance of items, the possibility of ambiguity and inadequate interpretations of phrases, or the presence of inadequacy in the meanings of words were examined. In the next stage, the quantitative method of the impact method was also used. Accordingly, the questionnaire was completed by 20 people and based on the participants' answers to the 5‐point Likert scale for each of the instrument questions, face validity was assessed quantitatively. For quantitative analysis of content validity, two indicators, content validity ratio (CVR) and content validity index (CVI), were used. The final scale achieved CVR = 0.78 and CVI = 0.84. According to Lawshe's table for 12‐panel experts, the critical CVR value was 0.56; therefore, all items with CVR ≥ 0.56 were retained. To determine the reliability of the final version, the internal consistency of the questionnaire was performed using the two‐half structural method and through Cronbach's alpha (*α* = 0.82). The questionnaire was pilot tested on a separate sample of 30 infertile couples (not included in the main study) to assess readability, clarity, and test‐retest reliability. The intraclass correlation coefficient (ICC) for test–retest reliability was 0.85 (95% CI: 0.78–0.90), indicating good temporal stability. Construct validity was not assessed using factor analysis, which is acknowledged as a limitation of the questionnaire development process. The instrument was used primarily for descriptive group comparisons rather than latent construct measurement. Finally, the questions used in this questionnaire included the sections on awareness of the law (7 questions), importance of this law (3 questions), and following this law (4 questions). Each item was rated on a 5‐point Likert scale. For most items, options ranged from 1 (very low) to 4 (very high), while option 5 was labeled as “not applicable” or varied based on the specific item content (e.g., “I don't know” for awareness items, “not relevant” for importance items). This variation was necessary to accommodate different response formats across the three domains (awareness, importance, follow‐up).

### 2.7 Data Collection Process

After obtaining permission from the Ethics Committee of Tabriz University of Medical Sciences and making the necessary coordination with the health centers that are the main providers of infertility services, couples completed questionnaires with their consent. It also tried to ensure that there was diversity in the selection of centers in terms of ownership type (government, private, military, etc.). Trained interviewers were used to collect the questionnaires.

This study was conducted in accordance with the ethical principles of the Declaration of Helsinki. The study protocol was reviewed and approved by the Ethics Committee of Tabriz University of Medical Sciences (Approval ID: IR. TBZMED. REC.1402.931). Informed consent was obtained from all adult participants after explaining the study aims. Participants were informed of their right to withdraw from the study at any time without consequences. Confidentiality of participants' data was ensured through anonymization and secure data handling.

### 2.8 Data Analysis Method

SPSS STATISTICS version 16 and STATA version 17 software were used to analyze the data. In the descriptive section, frequency (percentage) was used for qualitative variables, and mean, minimum, and maximum were used for quantitative variables. The normality of the data was checked using the Kolmogorov–Smirnov test, and due to the non‐normality of the data, non‐parametric Mann–Whitney *U* and Kruskal–Wallis tests were used to compare the means of the groups.

Spearman correlation analysis was also used to examine correlations. The significance level was also considered, *p* < 0.05. Finally, STATA software was used to perform multiple linear regression (Backward) and predict the effect of variables on infertility stress. In the Backward method, all variables are first entered into the model, then variables with a weaker significance level (*p*‐value > 0.10) are gradually removed until the final model contains only significant variables. Multicollinearity was assessed using the variance inflation factor (VIF), with VIF > 5 considered indicative of problematic collinearity. All VIF values in the final model were < 3, indicating no substantial multicollinearity. The backward elimination removal criterion was set at *p* > 0.10.

Model assumptions were checked. Normality of residuals was assessed using the Shapiro‐Wilk test and visual inspection of Q—Q plots. Homoscedasticity was examined using residual‐versus‐fitted plots. Multicollinearity was assessed using the variance inflation factor (VIF), with VIF > 5 indicating problematic collinearity. All VIF values in the final model were < 3.

Statistical terms and abbreviations: In this study, the following abbreviations were used: SD (standard deviation), CI (confidence interval), and VIF (variance inflation factor). All statistical tests were two‐sided. The a priori significance level was set at *α* = 0.05.

Pre‐specified versus exploratory analyses: The primary analyses (descriptive statistics, group comparisons using Mann–Whitney *U* and Kruskal–Wallis tests, and Spearman correlation) were pre‐specified. The multiple linear regression with backward elimination was exploratory, aimed at identifying potential predictors of infertility stress without pre‐specified hypotheses about specific variables. Subgroup analyses by sex, education level, and job status were exploratory and should be interpreted with caution.

Software and guidelines: Data were analyzed using IBM SPSS Statistics version 16 (IBM Corp., Armonk, NY, USA) and STATA version 17 (StataCorp LP, College Station, TX, USA). This study followed the SAMPL (Statistical Analyses and Methods in the Published Literature) guidelines for reporting statistical results.

The design of this cross‐sectional study follows the STROBE (Strengthening the Reporting of Observational Studies in Epidemiology) guidelines [23]. Statistical reporting follows the SAMPL guidelines as stated above [24].

## 3 Results

396 people participated in this study (response rate 94.2%) with an average age of 34.37 years. The minimum age of the participants in the study was 17 and the maximum was 54 years. Also, the average duration of marriage of the participants in the study was 7.95. The minimum duration of marriage was 1 and the maximum was 30 years. Other demographic characteristics of the participants are reported in Table 1.

**Table 1:** Demographic characteristics of participants in the study measuring the relationship between stress caused by infertility and awareness, importance, and follow‐up of receiving benefits from the “Support for the Family and Youth of the Population” law among infertile couples: in East Azerbaijan province, Iran.

Variables	Variable Levels	*N* (%)	Variables	Variable Levels	*N* (%)
Gender	Male	87 (22.4)	Housing situation	Yes	165 (43.7)
	Female	302 (77.6)		No	213 (56.3)
Education	Diploma	254 (66.7)	Place of residence	City	290 (76.9)
	Bachelor	91 (23.9)		Village	87 (23.1)
	Master	27 (7.1)	Religion	Shia	337 (92.1)
	Doctorate	9 (2.4)		Sunni	24 (6.6)
Job	Unemployed	43 (11.1)		Other	5 (1.4)
	Housekeeper	223 (57.6)	Economic situation	High	4 (1)
	Government employee	26 6.7)		Good	35(9)
	Non‐government employee	15 (3.9)		Average	186 (48.1)
	Freelance job	70 (18.1)		Low	131 (33.9)
	Other	10 (2.6)		Very low	31 (8)

Table 2 shows the descriptive indices of the main variables of the study, including infertility stress and other related variables in a sample of 396 infertile individuals. As can be seen, the average total infertility stress score is (169.78), and the lowest average stress score (9.01) is related to the follow‐up variable.

**Table 2:** Descriptive results of infertility stress and related variables among infertile couples in East Azerbaijan province, Iran.

Domain	Variables	*N*	Min	Max	Mean	SD	Scores Range
Fertility stress	Overall Fertility Stress	396	91	250.44	169.78	29.98	[46–276]
	Social Concerns	396	15	54.44	32.41	7.15	[10–60]
	Sexual Concerns	390	8	48	27.31	9.73	[8–48]
	Relationship Concerns	383	10	60	34.36	9.49	[10–60]
	Rejection of Child‐free Lifestyle	377	12	48	31.52	7.82	[8–‐48]
	Need for Parenthood	374	15	60	45.54	10.74	[10–60]
“Support for the Family and Youth of the Population” law	Knowledge	393	7	35	17.61	7.13	[7–35]
	Importance	391	3	15	10.32	4.11	[3–15]
	Follow‐up	393	3	15	9.01	3.65	[3–15]

The results in Table 3 demonstrate significant associations between demographic variables and infertility‐related stress/concerns. Women exhibited higher infertility stress compared to men, whereas men reported greater social, sexual, and childbearing concerns. These comparisons are descriptive and based on raw mean differences. Effect sizes (e.g., Cohen's d) were not calculated, and no interaction tests (e.g., gender × other variables) were performed due to the exploratory nature of the subgroup analyses. Therefore, the observed differences should be interpreted with caution.

**Table 3:** Results of measuring the relationship between demographic variables, infertility stress and other related variables among infertile couples in East Azerbaijan province, Iran.

Variables	Variable Levels	Overall Fertility Stress		Knowledge		Importance		Follow‐up	
		Mean ± SD	*p* value	Mean ± SD	*p* value	Mean ± SD	*p* value	Mean ± SD	*p* value
Sex	Male	163.87 ± 32.62	*p* = **0.004**	16.83 ± 6.96	*p* = 0.304	10.4 ± 4.45	*p* = 0.685	8.95 ± 3.8	*p* = 0.588
	Female	171.27 ± 29.27		17.74 ± 7.23		10.3 ± 4.03		9.03 ± 3.6	
Education	Diploma	172.43 ± 28.26	*p* = **0.003**	17.09 ± 7.58	*p* = 0.189	10.21 ± 4.43	*p* = 0.135	9.05 ± 3.92	*p* = 0.091
	Bachelor	165.68 ± 31.09		18.3 ± 6.28		10.3 ± 3.42		8.74 ± 3.15	
	Master	168.15 ± 33.14		19.82 ± 5.87		11.33 ± 2.89		10.11 ± 2.4	
	Doctorate	139.47 ± 30.9		15.66 ± 6.91		8 ± 3.27		6.33 ± 3.08	
Job	Unemployed	160.79 ± 38.51	*p* < **0.001**	16.72 ± 6.63	*p* = **0.002**	8.8 ± 3.83	*p* = **0.017**	7.81 ± 3.13	*p* = **0.016**
	Housekeeper	174.84 ± 25.9		17.61 ± 7.36		10.25 ± 4.25		9.11 ± 3.73	
	Government employee	168.57 ± 26.69		22.74 ± 4.84		12.36 ± 1.86		10.96 ± 2.27	
	Non‐government employee	158.12 ± 32.93		16.05 ± 5.05		10.2 ± 3.25		7.73 ± 2.89	
	Freelance Job	165.29 ± 32.58		16.46 ± 6.81		10.44 ± 4.26		8.86 ± 3.85	
	Other	143.88 ± 32.63		16.9 ± 7.7		11.55 ± 4.05		8.2 ± 3.08	
Housing situation	Yes	166.91 ± 29.58	*p* = 0.15	17.54 ± 6.97	*p* = 0.081	10.3 ± 3.91	*p* = 0.378	8.99 ± 3.34	*p* = 0.903
	No	172.67 ± 30.26		17.29 ± 7.24		10.26 ± 4.28		8.92 ± 3.89	
Place of residence	City	168.48 ± 30.39	*p* = 0.201	17.69 ± 7.03	*p* = 0.285	10.4 ± 4.04	*p* = 0.446	8.92 ± 3.59	*p* = 0.442
	Village	173.87 ± 28.1		17.32 ± 7.68		10 ± 4.18		9.16 ± 3.85	
Religion	Shia	170.38 ± 29.71	*p* = 0.941	17.58 ± 7.16	*p* = 0.484	10.44 ± 4.05	*p* = 0.421	9.01 ± 3.65	*p* = 0.758
	Sunni	166.26 ± 28.08		16.72 ± 7.15		9.2 ± 4.58		8.7 ± 3.98	
	Other	151.53 ± 22.75		24.8 ± 5.67		13.7 ± 1.98		12.2 ± 3.03	
Economic situation	High	134.08 ± 31.46	*p* = 0.208	22.25 ± 11.87	*p* = 0.179	10 ± 6	*p* = 0.818	8.75 ± 5.05	*p* = 0.394
	Good	165.99 ± 26.55		19.95 ± 7.87		9.7 ± 4.3		8.34 ± 3.52	
	Average	165.24 ± 30.86		17.3 ± 6.6		10.39 ± 3.87		9.05 ± 3.53	
	Low	175.54 ± 28.09		17.77 ± 7.33		10.6 ± 3.99		9.3 ± 3.65	
	Very low	180.64 ± 30.83		15.1 ± 6.97		9.4 ± 5.26		8.15 ± 4.11	

Variables	Variable levels	Social concerns		Sexual concerns		Relationship concerns		Rejection of a child‐free lifestyle		Need for parenthood	
		Mean ± SD	*p* value	Mean ± SD	*p* value	Mean ± SD	*p* value	Mean ± SD	*p* value	Mean ± SD	*p* value
Sex	Male	32.10 ± 11.05	*p* = **0.044**	27.01 ± 8.96	*p* = **0.015**	30.82 ± 9.86	*p* = 0.108	32.43 ± 9.7	*p* = **0.019**	26.88 ± 11.92	*p* = **0.002**
	Female	29.36 ± 10.2		23.67 ± 9.02		29.28 ± 9.89		29.77 ± 11.52		22.62 ± 10.7	
Education	Diploma	29.12 ± 10.63	*p* = **0.004**	22.99 ± 8.58	*p* < **0.001**	28.54 ± 9.46	*p* = **0.01**	29.99 ± 10.73	*p* = 0.357	22.68 ± 10.6	*p* = **0.013**
	Bachelor	30.73 ± 10.14		27.07 ± 9.62		31.2 ± 10.5		29.66 ± 12.69		24.54 ± 11.55	
	Master	29.84 ± 9.11		26.12 ± 8.4		33.12 ± 8.76		33.56 ± 10.3		22.64 ± 10.15	
	Doctorate	40 ± 6.51		30 ± 9.69		29.22 ± 12.07		31.22 ± 8.77		34.44 ± 13.29	
Job	Unemployed	35.24 ± 9.84	*p* = **0.001**	28.2 ± 8.25	*p* < **0.001**	33.39 ± 8.01	*p* = **0.005**	32.15 ± 8.86	*p* = 0.403	31.17 ± 14.25	*p* < **0.001**
	Housekeeper	29.22 ± 9.92		22.89 ± 8.65		28.89 ± 9.34		30.88 ± 10.78		21.9 ± 9.43	
	Government Employee	29.04 ± 9.3		25.64 ± 7.15		33.04 ± 9.07		34.36 ± 14.54		20.72 ± 7.08	
		32.17 ± 10.52		25.08 ± 8.27		29.8 ± 9.98		25.92 ± 10.49		26.92 ± 11.24	
	Freelance Job	30.85 ± 11.61		26.7 ± 9.05		30.25 ± 10.37		29.1 ± 10.83		24.54 ± 11.04	
	Other	35.75 ± 10.47		33.88 ± 6.99		30. 5 ± 13.99		26.75 ± 12.38		19.75 ± 14.43	
Housing situation	Yes	31.93 ± 10.11	*p* = **0.013**	25.18 ± 9.28	*p* = 0.219	30.3 ± 9.13	*p* = 0.467	31.41 ± 10.28	*p* = 0.088	24.42 ± 11.36	*p* = 0.236
	No	29.31 ± 10.41		24.4 ± 8.45		29.67 ± 10		29.86 ± 11.53		23.35 ± 10.71	
Place of residence	City	30.58 ± 10.21	*p* = 0.160	25.15 ± 9.05	*p* = 0.217	30.16 ± 9.07	*p* = 0.118	30.73 ± 11	*p* = 0.497	24.25 ± 11.36	*p* = 0.306
	Village	30.17 ± 10.9		23.23 ± 7.85		29.17 ± 9.28		29.93 ± 10.99		22.27 ± 9.48	
Religion	Shia	30.48 ± 10.34	*p* = 0.749	24.53 ± 8.82	*p* = 0.154	29.82 ± 9.74	*p* = 0.498	30.62 ± 11.22	*p* = 0.639	23.72 ± 10.97	*p* = 0.275
	Sunni	29.27 ± 10.51		28.95 ± 9.4		31.27 ± 8.76		30.14 ± 8.51		24.05 ± 11.4	
	Other	33.8 ± 10.35		22.2 ± 5.41		31.3 ± 7.75		29.8 ± 9.33		27 ± 12.08	
Economic situation	High	35.67 ± 9.29	*p* = 0.228	29.67 ± 8.73	*p* = **0.001**	41 ± 6.55	*p* = 0.007	33.67 ± 7.63	*p* = 0.143	25 ± 18.02	*p* = **0.032**
	Good	31.1 ± 9.89		28.27 ± 8.33		35 ± 7.24		31.93 ± 8.18		24.8 ± 9.22	
	Average	31.46 ± 9.9		26.23 ± 8.78		30.13 ± 9.28		29.86 ± 11.29		25.38 ± 11.21	
	Low	29.17 ± 10.05		22.54 ± 7.6		28.86 ± 9.76		30.01 ± 11.16		21.55 ± 9.68	
	Very low	29.19 ± 14.01		21 ± 11.28		26.78 ± 10.99		35.26 ± 10.73		23.26 ± 14.8	

*Note:* Bold values indicate statistically significant.

Education level showed statistically significant correlations with infertility stress, social/sexual/relationship concerns, and childbearing needs. Occupational status significantly influenced multiple variables: government employees displayed the highest awareness scores, while unemployed participants had the highest concern levels and lowest compliance scores across domains (knowledge, importance, pursuit, social/sexual/relationship concerns, and childbearing needs). Improved economic status correlated with increased sexual concerns and childbearing needs. Housing status was correlated with higher infertility stress scores.

Table 4 shows the correlation results. As is clear, infertility stress is significantly associated with all variables studied except age and duration of marriage, with the highest correlation observed with sexual (*r* = 0.74) and relationship (*r* = 0.73) concerns (*p* < 0.01). Age was also associated with awareness and follow‐up (*r *= 0.14).

**Table 4:** Results of the study of the correlation test of infertility stress and other related variables among infertile couples in East Azerbaijan province, Iran.

Variables	1	2	3	4	5	6	7	8	9	10	11
1. Overall fertility stress	_										
2. Knowledge	0.36**	_									
3. Importance	0.31**	0.56**	_								
4. Follow‐up	0.37**	0.58**	0.70**	_							
5. Social concerns	0.68**	0.10*	0.08	0.11*	_						
6. Sexual concerns	0.74**	0.08	0.05	0.15**	0.54**	_					
7. Relationship concerns	0.73**	0.03	−0.01	0.11*	0.46**	0.65**	_				
8. Rejection of child‐free lifestyle	0.47**	0.08	0.00	0.02	0.17**	0.17**	0.33**	_			
9. Need for parenthood	0.68**	0.08	0.16**	0.17**	0.48**	0.46**	0.49**	0.10	_		
10. Age	0.05	0.14**	0.07	0.14**	0.02	−0.08	0.05	0.13*	0.03	_	
11. Marriage	−0.04	−0.03	−0.06	−0.04	0.02	−0.02	−0.03	0.09	−0.03	0.19**	_

*Note:* All correlation coefficients (*r*) are Spearman's ρ.

* *p* < 0.05

** *p* < 0.01 (two‐tailed). Key correlations with 95% confidence intervals: sexual concerns (*r* = 0.74, 95% CI: 0.69–0.78), relationship concerns (*r* = 0.73, 95% CI: 0.68–0.77), social concerns (*r* = 0.68, 95% CI: 0.62–0.73), need for parenthood (*r* = 0.68, 95% CI: 0.62–0.73).

Table 5 shows the results of the multiple linear regression analysis using the Backward method. Unstandardized coefficients (B) are reported along with their 95% confidence intervals. The final model explained a substantial proportion of the variance in infertility stress (*R* ^2^ = 0.9543, adjusted *R* ^2^ = 0.9521). Demographic variables including age, education, job status, economic status, housing, and place of residence were entered as candidate predictors; those with *p* > 0.10 were removed during backward elimination. In this model, the strongest predictors of infertility stress were the need to have children (B = 2.579), relationship concerns (B = 1.464), and social concerns (B = 1.254), respectively. Higher education (codes 4 and 5) also increased stress, while some jobs (codes 2, 3, and 6) had a reducing effect.

**Table 5:** Results of multiple linear regression of effective variables of infertility stress among infertile couples in East Azerbaijan province, Iran.

Variables	B (unstandardized)	Std. err	*t*	*p* value	95% CI	
Rejection of child‐free lifestyle	0.866	0.048	17.82	0.000	0.77	0.961
Social concerns	1.254	0.057	22	0.000	1.142	1.366
Relationship concerns	1.464	0.069	21.03	0.000	1.327	1.601
Sexual concerns	1.037	0.08	12.94	0.000	0.879	1.195
Need for parenthood	2.579	0.138	18.64	0.000	2.307	2.851
Knowledge	0.905	0.093	9.67	0.000	0.721	1.089
Importance	1.346	0.194	6.91	0.000	0.962	1.729
Follow‐up	1.166	0.22	5.28	0.000	0.732	1.601
Education						
3	8.464	3.075	2.75	0.006	2.411	14.518
4	18.026	8.381	2.15	0.032	1.526	34.526
Job						
2	−2.803	1.138	−2.46	0.014	−5.044	−0.562
3	−6.174	1.993	−3.1	0.002	−10.098	−2.25
6	−9.686	3.555	−2.72	0.007	−16.68	−2.687
_cons	4.357	3.015	1.45	0.150	−1.579	10.293

*Note:* All coefficients are unstandardized. Standardized coefficients (β) are available upon request.

N: 288.

R^2^: 0.9543.

## 4 Discussion

This study investigated the relationship between infertility‐related stress and awareness of facilities provided by the “Support for the Family and Youth of the Population” law among infertile couples in East Azerbaijan Province. Figure 1 presents the conceptual framework guiding this study. Our findings suggested that the participants' mean infertility stress score was 169.78, with the highest correlations observed for sexual (*r* = 0.74) and relationship (*r* = 0.73) concerns. Women experienced higher overall stress, while men reported greater social and sexual concerns. Furthermore, higher education levels were associated with lower stress, and government employment correlated with greater awareness of the law's provisions. Remarkably, increased awareness and pursuit of legal facilities were positively associated with higher stress levels, which may reflect unrealistic expectations and administrative hurdles.

Importantly, the finding that men reported higher social and sexual concerns despite having lower total stress scores may reflect gender differences in how stress is experienced and expressed. However, as these comparisons were exploratory and effect sizes were not formally compared, these results should be considered hypothesis‐generating rather than confirmatory.

The observed gender differences in infertility‐related stress must be interpreted within Iran's specific cultural context. In Iranian society, motherhood is deeply intertwined with female identity, social worth, and family honor. As previously reported in Iranian samples [25]. Women often face pronounced pressure from extended family members (particularly mothers‐in‐law), intrusive questions about childbearing, and social stigma if infertility is perceived as “female responsibility.” This cultural backdrop explains why women in our study reported higher overall infertility stress compared to men. Conversely, men expressed greater social and sexual concerns. This seemingly paradoxical finding may reflect that Iranian men, as primary breadwinners, face distinct pressures. First, social concerns relate to perceived failure in fulfilling family continuation expectations and potential inheritance disruptions. Second, sexual concerns may stem from cultural scripts linking male potency to fertility, where infertility threatens masculine identity and sexual self‐efficacy. Consistent with gender‐specific coping patterns [26], men typically have fewer emotional support networks than women in Iranian culture, as help‐seeking is often stigmatized as weakness. These culturally‐shaped gender dynamics imply that interventions may need to be gender‐sensitive: women may benefit from counseling that addresses social stigma and family pressures, while men may require interventions focusing on sexual health communication and expanding emotional support networks. Moreover, cultural stigma surrounding infertility may also discourage couples from actively pursuing legal support services, as doing so might publicly disclose their condition. This cultural barrier could further explain why awareness of facilities is not always associated with lower stress.

These findings align with a recent comprehensive review by Ribeiro Neto et al. [27], which emphasizes that infertility extends beyond medical aspects to include emotional, social, and ethical complexities. The review supports our conclusion that supportive policies should integrate mental health services alongside financial incentives.

The results of the current study align with those of El Kissi et al. in Tunisia [28] and Gourounti et al. in Greece [29], in that women generally experience markedly higher infertility‐related stress than men. Conversely, our study showed that men expressed greater concerns across regarding social dynamics, sexuality, and childbearing aspirations, compared to women. This finding contradicts the results of Chehreh et al. in Iran [25], Karaca et al. in Turkey [30], and Wang et al. in Taiwan [31]. These earlier studies suggested that children are considered the outcome of marriage and are always a social expectation, with familial attitudes, interpersonal interactions, and societal pressures being major factors influencing perceived stress in infertile women. Nevertheless, it seems that infertile couples are constantly striving to cope with these stresses, and due to gender differences, the methods and approaches to managing this issue vary between women and men [26].

Another notable finding was the considerable association between education level and both the extent of infertility‐related stress and its associated concerns. Our results revealed that a higher education level (above diploma) was significantly associated with lower infertility stress among couples compared to those with a diploma or lower education. This finding is consistent with Newton et al. in Canada [20] and Lei et al. in China [32]. However, it contradicts the study by Wiweko et al. in Indonesia [33], which found no significant relationship between education and fertility stress. Possible reasons for the association between higher education and lower infertility stress include greater access to information and support resources, better problem‐solving and stress management skills, higher income levels, and stronger social networks, all of which can mitigate the financial and psychological burden of infertility. Given that higher education acts as a protective factor against infertility stress, policymakers could implement targeted interventions (educational, supportive, and economic) focusing on equitable access to information and the provision of mental health and treatment services to reduce this gap in less educated groups.

The results of this study also indicate that improved economic conditions show dual associations with couples' outcomes. While increased financial capacity is related to a stronger desire to have children, as it fosters a sense of security and readiness for parental responsibilities. This improved economic status is associated with increased sensitivity regarding sexual and marital dynamics, potentially giving rise to new concerns. These findings suggest that while improved financial status is generally considered a positive factor, it can also create specific challenges in the realm of fertility. This underscores the importance of simultaneously considering economic and psychosocial factors in fertility‐related planning and highlights that supportive interventions must pay special attention to these complexities.

Employment status emerged as a critical determinant of infertility experiences, aligning with prior studies [34, 35]. Occupation type correlated significantly not only with infertility‐related stress but also with treatment awareness, attitudes, and compliance. Public sector employees had superior knowledge about infertility and the Family and Population Protection Law compared to other groups, likely associated with workplace communication networks and policy‐linked benefits (e.g., leave, loans). These findings suggest that supportive and educational interventions for infertile couples should be designed and implemented with consideration for their employment status and specific circumstances to maximize effectiveness. One reason for the low awareness and lack of follow‐up among other segments of society might be the weakness of information dissemination and public awareness campaigns through media outlets like television. Based on the literature review and the research team's experiences, very limited information is available regarding the process and formulation of this policy. Therefore, The Iranian Broadcasting Corporation and municipal agencies must prioritize both disseminating high‐quality information about the law's provisions and increasing public trust in their implementation [36].

Correlation analyses showed a positive and significant correlation between infertility‐related stress and most research variables, except age and duration of marriage, which did not show a significant association. The current study found a strong correlation between infertility stress and sexual concerns (*r* = 0.74) and communication concerns (*r* = 0.73). It should be noted that the high correlations between total infertility stress and its subscales (sexual and relationship concerns) are partly attributable to part‐whole relationships, as the subscales are components of the total score. A multidimensional analysis of these findings suggests that multiple factors contribute to this relationship. On one hand, the inherent sensitivity of sexual issues in infertility including performance anxiety, reduced sexual satisfaction, and the transformation of marital relations into a “duty” under social pressures, and on the other hand, communicative tensions arising from marital conflicts, gender differences in coping styles, and lack of social support may be partly explained by cultural stigma, and all these factors are correlated with higher stress levels. To effectively address these complex challenges, comprehensive strategies are proposed, including specialized psychological interventions (such as emotionally‐focused couples therapy and sex therapy), skill‐based educational programs, development of peer support networks, and health‐oriented policies (such as expanding insurance coverage for psychological services and implementing organizational support programs). This integrated approach, which simultaneously addresses individual, interpersonal, and socio‐cultural dimensions, can lead to a significant improvement in the quality of life for infertile couples by reducing stressors and strengthening coping mechanisms. Its success, however, requires interdisciplinary cooperation among gynecologists, health psychologists, and health policymakers, while considering the local characteristics of each community.

The positive association between awareness of legal facilities and infertility‐related stress can be understood through established theoretical frameworks. According to Lazarus and Folkman's Stress and Coping Theory [37], individuals continuously appraise situations as threats or challenges. When infertile couples become aware of legal facilities but encounter administrative barriers, this situation may be appraised as a threat (unmet need) rather than a challenge, which may be associated with increased stress. Additionally, Expectation Disconfirmation Theory [38] suggests that satisfaction (or distress) is related to the discrepancy between expectations and actual outcomes. Information dissemination about the law may be associated with positive expectations of support; when couples face bureaucratic complexities and delays, the negative disconfirmation between expected and experienced support paradoxically is associated with greater distress. These theoretical perspectives support our empirical finding that awareness is associated with higher stress levels rather than lower stress.

According to the findings of the current study, increased awareness, importance, and pursuit of family and population support facilities showed a positive correlation with infertility stress in couples. This suggests that rather than reducing infertility stress, it was correlated with higher stress levels. Several reasons could explain this. First, there's a gap between the expectations created by information dissemination and the realities of implementation, including complex administrative processes, extensive documentation requirements, and lengthy delays in receiving facilities, which is correlated with disappointment and increased psychological tension. Second, the additional burden of continuously pursuing these facilities within bureaucratic administrative systems, especially for couples simultaneously undergoing infertility treatments, is associated with higher reported stress. Third, the direct and indirect costs of pursuing these supports (including transportation and documentation expenses) are related to an additional financial burden. Eventually, the sole focus of support systems on material aspects and neglect of specialized psychological support makes it difficult to manage expectations and disappointments. To address these challenges, practical solutions are proposed, including simplifying administrative processes, developing electronic tracking systems, providing simultaneous psychological counseling, training administrative staff for more sensitive interactions, and establishing special support hotlines to answer questions and address couples' problems. These findings indicate the indispensable necessity of designing comprehensive support systems that simultaneously address the material and psychosocial dimensions of infertility.

Furthermore, the results of multivariate regression analysis identified that among the predictor variables, the need for parenthood with the highest standard coefficient (B = 2.579) had the strongest statistical association with stress, which underscores the importance of the desire to have children in the experience of infertility‐related stress. After that, relationship concerns (B = 1.464) and social concerns (B = 1.254) had the greatest contribution to predicting stress. This finding is crucial from several perspectives. On one hand, in many cultures, childbearing is considered part of social identity and individual development, and family and social pressures can transform this need into a psychological compulsion. On the other hand, infertility can lead to a sense of inadequacy in gender and parental roles, as for many couples, childbearing is an essential part of life planning and the realization of shared aspirations. Moreover, the stronger the desire for children, the more stressful the encounter with infertility will be, especially when this need is intertwined with religious beliefs or an individual's life philosophy.

Remarkably, the variables “importance” (B = 1.346), “follow‐up” (B = 1.166), and “awareness” (B = 0.905) all were positively correlated with stress. The regression results also were consistent with the correlation findings, further showing increased awareness and pursuit of supportive facilities were associated with higher stress levels rather than a reduction. Conversely, certain occupations (government employees and homemakers) showed a stress‐reducing effect. These results emphasize the importance of considering individual differences in supportive interventions.

## 5 Limitations

Despite providing valuable insights into the relationship between infertility stress and related factors, this study has limitations that should be considered when interpreting the results. These limitations include the geographic focus of the study on East Azerbaijan Province, which may influence the generalizability of the findings to the broader population. Furthermore, the cross‐sectional design strictly precludes any causal inference. Additionally, as noted in the Methods section, the unit of analysis was the individual participant, and intra‐couple dependency could not be modeled due to the absence of matched data for both partners. This limits our ability to examine dyadic effects. Additionally, the reported correlations between total infertility stress and its subscales (e.g., sexual and relationship concerns) are subject to part‐whole bias, as the subscales are mathematical components of the total score. This inflation should be considered when interpreting the magnitude of these coefficients. Future studies should report correlations among subscales separately to avoid this overlap. Other limitations include the lack of control for certain potential confounding variables, such as psychiatric history, marital quality, and unmeasured influential variables. Additionally, the use of self‐reported data may introduce social desirability bias, as participants might underreport or overreport certain responses (e.g., sexual concerns or perceived importance of legal facilities) based on perceived social norms.

Furthermore, subgroup comparisons by gender were exploratory, and no formal interaction tests or effect size comparisons (e.g., Cohen's *d*) were conducted. Therefore, the observed gender differences should be interpreted with caution. Future studies should test gender‐by‐stress interactions using confirmatory approaches. In spite of these limitations, which emphasize the need for future studies with more diverse samples, longitudinal designs, qualitative approaches, and mixed methods, the present findings can serve as a valuable foundation for future research and the design of clinical interventions.

## 6 Conclusion

The present study, while offering valuable insights into factors influencing infertility stress, specifically demonstrated that awareness and pursuit of benefits under the “Support for the Family and Youth of the Population” law were positively associated with infertility‐related stress, rather than being associated with reduced stress. This finding likely reflects implementation challenges rather than the intent of the policy.

The observed positive association may stem from gaps between policy promises and implementation realities. Extensive information dissemination about facilities may create unrealistic expectations, while bureaucratic complexities and payment delays are associated with a higher psychological burden, potentially creating a cycle of disappointment and feelings of failure.

These findings suggest that the implementation of current policies may not have fully accounted for the psychological realities and administrative burdens faced by infertile couples. This underscores the necessity of addressing implementation gaps in current policies, including simplifying administrative processes, managing expectations through realistic awareness campaigns, and integrating psychological support alongside financial incentives. Such an approach might help transform these policies into a source of relief rather than an additional burden.

## Author Contributions

**Saber Azami‐Aghdash:** writing – original draft, writing – review and editing, methodology. **Ramin Rezapour:** formal analysis, writing – original draft. **Samin Banaei Rezaeiyeh:** data curation. **Zahra Vakilazad Sarabi:** data curation. **Leyla Najafi:** data curation. **Mohsen Nouri:** data curation. **Esmail Ezzati:** supervision, writing – review and editing, formal analysis, project administration. All authors read and approved the final manuscript and agreed to be accountable for all aspects of the work in ensuring that questions related to the accuracy or integrity of any part of the work are appropriately investigated and resolved.

## Funding

This research did not receive any specific grant from funding agencies in the public, commercial, or not‐for‐profit sectors. The supporting sources had no involvement in study design; collection, analysis, and interpretation of data; writing of the report; or the decision to submit the report for publication.

## Ethics Statement

Details of ethical approval and informed consent are provided in the Methods section (Data collection process). The study protocol was reviewed and approved by the Ethics Committee of Tabriz University of Medical Sciences (Approval ID: [IR. TBZMED. REC.1402.931]).

## Conflicts of Interest

The authors declare no conflicts of interest.

## Use of Artificial Intelligence (AI)

We have not used any AI tools or technologies to prepare this manuscript.

## Transparency Statement

The lead author Esmail Ezzati affirms that this manuscript is an honest, accurate, and transparent account of the study being reported; that no important aspects of the study have been omitted; and that any discrepancies from the study as planned (and, if relevant, registered) have been explained.

## Data Availability

The data that support the findings of this study are available from the corresponding author (Esmail Ezzati, e.ezati4770@gmail.com) upon reasonable request. Due to privacy and ethical restrictions (participant confidentiality under approval ID IR. TBZMED. REC.1402.931), data are not publicly available.
